# Tissue Transparency In Vivo

**DOI:** 10.3390/molecules24132388

**Published:** 2019-06-28

**Authors:** Mikhail Inyushin, Daria Meshalkina, Lidia Zueva, Astrid Zayas-Santiago

**Affiliations:** 1 Department of Physiology, Universidad Central del Caribe, Bayamon, PR 00960, USA; 2 Sechenov Institute of Evolutionary Physiology and Biochemistry, St. Petersburg 194064, Russia

**Keywords:** in vivo transparency, imaging depth, optical tissue clearing, deep-tissue optogenetics, genetically modified animals, model organisms with transparent tissues

## Abstract

In vivo tissue transparency in the visible light spectrum is beneficial for many research applications that use optical methods, whether it involves in vivo optical imaging of cells or their activity, or optical intervention to affect cells or their activity deep inside tissues, such as brain tissue. The classical view is that a tissue is transparent if it neither absorbs nor scatters light, and thus absorption and scattering are the key elements to be controlled to reach the necessary transparency. This review focuses on the latest genetic and chemical approaches for the decoloration of tissue pigments to reduce visible light absorption and the methods to reduce scattering in live tissues. We also discuss the possible molecules involved in transparency.

## 1. Introduction

Many animals have transparent tissues or are completely transparent in their everyday life. Some can even become entirely transparent at will, such as certain cephalopod mollusks that change the light adsorption of their skin (Zylinski and Johnsen, 2011) [1], depending on pictorial depth cues and directional illumination (Zylinski et al., 2016) [2]. Animals with natural transparency have significant advantages for research, extending the range of possibilities for the application of different optical methods (Fetcho and O’Malley, 1995; O’Malley et al., 2003; White et al., 2008; Bin and Lyons, 2016; Antinucci and Hindges, 2016; Harrison et al., 2016; Nicolson, 2017; Saleem and Kannan, 2018) [3,4,5,6,7,8,9,10]. Even if the animal is not generally transparent, it usually has specialized living transparent cells, at least in the visual system of the animal, and this cell transparency can be specifically analyzed (Zayas et al., 2018) [11]. Questions arise: Why are some living cells transparent, while others are not? Can we maintain living cells while making them transparent in vivo for technical purposes? Can we make animals transparent or at least partially transparent or develop transparent animal tissues and organs in vivo?

The classical view is that a tissue is transparent if it neither absorbs nor scatters light. Thus, absorption and scattering are the key elements to be controlled to reach the necessary tissue transparency.

Except for a few pigments, the majority of organic molecules in the cell have no ability to absorb visible light. Thus, (1) elimination of these pigments will reduce absorption and thereby yield decoloration. This can be achieved chemically by addition of reactive substances that eliminate specific pigments or genetically by identifying specific mutations that disrupt biosynthesis of the pigment. After elimination of pigments, the primary reason for any remaining opacity of organic tissues is light scattering (Brunsting and Mullaney, 1974; Tardieu and Delaye, 1988) [12,13].

Light scattering is caused by localized nonuniformities in refractive indices (RIs, Jacques and Pogue, 2008; Jacques, 2013, Perelman, 2017) [14,15,16]. These nonuniformities lead to alteration of the direction of light propagation when they pass through the interface between regions of different RIs (e.g., cytoplasm–organelle–cytoplasm or cytoplasm–protein–cytoplasm). Given enough directional changes due to multiple instances of refraction and reflection, the tissue, though nonabsorbing, will be opaque and nontransparent (Johnsen, 2001; Zueva et al., 2016) [17,18]. To reduce this scattering, (2) chemical or genetic procedures can be applied to homogenize mismatched RIs between individual cellular organelles and the cytoplasm. Chemical substances may be injected into the cytoplasm, changing RIs of individual cellular elements or the cytoplasm, or the refractive substance may be generated inside the cytoplasm due to genetic alterations that change the cytoplasm or organelle RI. Of course, complete transparency, which is advantageous in predator–prey competitions, can only be attained with aquatic animals, because the RI of their natural habitat can be easily matched to the cytoplasm RI. This complete transparency is difficult to achieve in animals that live in air or on land, and these animals usually have few transparent tissues. In addition, the interference of cellular elements plays a role in opacity, which to some extent can be managed by homogenizing mismatched RIs and reducing optical edges. In addition, transparency within the visible spectrum can be “partially restored” by newly developed digital microscopy methods, such as transmission matrix inversion (Xu et al., 2017; Cua et al., 2017) [19,20], which may also overcome light scattering problems.

## 2. Methods for Reducing Light Absorption

### 2.1. Chemical Decoloration of Pigments

The most common biomolecule causing chemical coloration and one of most important pigments in the animal world is melanin. Different melanin-type black pigments are formed through a series of oxidative reactions involving the amino acid tyrosine in the presence of the enzyme tyrosinase, and many substances that affect this synthesis can be used for skin depigmentation (Parvez et al., 2006; Heriniaina et al., 2018) [21,22]. Among the skin melanin lightening and depigmenting agents, magnesium-l-ascorbyl-2-phosphate (MAP), hydroxyanisole, *N*-acetyl-4-*S*-cysteaminylphenol, arbutin (hydroquinone-beta-d-glucopyranoside), and hydroquinone (HQ) as well as kojic, glycolic, and azelaic acids are the most widely known worldwide (reviewed in Parvez et al., 2006 [21]). In zebrafish larvae, an excellent vertebrate model for in vivo imaging because of its low level of light scattering, the most popular melanin decolorant is 1-phenyl-2-thiourea (Karlsson et al., 2001) [23]. Many other known pigments are the products of purine metabolism. For example, ptherines are usually synthesized from guanosine phosphate (Rebelo et al., 2003) [24], while many carotenoids (xanthines) are synthesized directly from guanine and are usually affected by different xanthine oxidases. Astaxanthin is the most frequent carotenoid in sea animals (Matthews et al., 2006) [25], and it was shown that blocking carotenoid synthesis by adrenocorticotropin (ACTH) reduces yellow-red coloration in goldfish (Hata and Hata, 1972) [26]. Carotenoids are also the main colorant in fish muscle cells. It is known that xanthine dehydrogenase oxidizes the yellow pteridine xanthopterin to the colorless pteridine leucopterin, and substances that affect this process also affect the chitin color in fly (Forrest et al., 1956) [27]. Many sea mollusks change the light absorption spectrum in different skin patches at will (Demski, 1994; Liuet al., 2017; Reiter et al., 2018) [28,29,30]. Similarly, rapid skin light absorption changes employing different specialized mechanisms are present in some anurans (Saenko et al., 2013; Teyssier et al., 2015) [31,32].

The pigment cells of cyprinid fishes, including zebrafish, comprise three types. First, there are the iridophores, which are packed with purine crystals identified as guanine and hypoxanthine. Iridophores in fish usually produce coloration due to the reflection or interference of light by regular alternating layers of guanine or hypoxanthine and cytoplasm, functioning as a Bragg-type light reflector (usually having the appearance of colored metal). In some fish species the spacing between layers can be changed by an internal cellular apparatus activated by a cellular light sensor, thus changing the wavelength of light that is most strongly reflected and shifting the iridophore color (Lythgoe et al., 1984; Schweikert et al., 2018) [33,34]. The second type of pigment cells are the melanophores (with black melanin in melanosomes), and the third type of pigment cells, found in zebrafish, are the xanthophores, which contain pteridine pigments (in pterinophores) and a small amount of carotenoid droplets. It has been suggested that the yellow color of xanthophores in zebrafish are mainly due to sepiapterin (Ziegler et al., 2000) [35]. The development of the pigmentation pattern in zebrafish is a tightly regulated process that depends on both the self-organizing properties of pigment cells and on extrinsic cues from other tissues (Eskova et al., 2017) [36]. Substances affecting pigmentation have been frequently investigated in zebrafish as the preferred animal model, with chemicals reducing coloration and augmenting the overall transparency of the larvae (Gunia-Krzyżak et al., 2016; Lajis, 2018) [37,38].

### 2.2. Genetic Approach to Removing Pigments

Many researchers worldwide use genetically selected albino animals that lack skin melanin. Albino animal skin is much more transparent; thus, these animals have extreme sun sensitivity, and even mechanosensitive neurons in the skin have become affected by light, impairing their functioning (Ono et al., 2017) [39]. However, the original reason for using albinos was as a genetic marker for perfectly inbred animals and to get pure animal lines (Rader, 2004) [40]. Interestingly, white people, with less melanin, historically populated the northern regions of Earth. Black people have less transparent skin, which absorbs more UV light with melanin than does white skin, and therefore require more sun exposure to produce the same amount of vitamin D. This led to the vital advantage of natural mutations that reduced skin coloration in people in northern regions and thereby reduced vitamin D deficiency (Conway and Trudeau, 2018) [41]. As we already mentioned, aquatic animals have an advantage if they are transparent, and they have many natural mutations that reduce skin pigmentation. Zebrafish that lack iridophores are known as roy orbison (roy) mutants, those that lack melanophores as albino mutants, and those that lack both melanophores and iridophores are known as ruby mutants. The zebrafish nacre mutant has no melanocytes, due to deletion of the melanocyte-inducing transcription factor (mitfa) gene as the result of genetic engineering (Lister et al., 1999) [42]. This mutant was crossed with the roy spontaneous mutant, generating the compound roy/nacre double homozygous mutant and resulting in a complete loss of the iridophore layer (White et al., 2007) [5]. The authors named this double mutant casper, referencing the famous cartoon ghost of the same name. Casper displays combined melanocyte and iridophore loss, in which the body of the fish is largely transparent due to loss of light absorption and reflection from iridiphores (White et al., 2007) [5]. Later, the roy mutation was found to be dependent on the mpv17 gene (D’Agati et al., 2017) [43]. Another clear zebrafish mutation named crystal was designed especially for in vivo imaging (Antinucci, Hindges, 2016) [7]. This mutant was obtained by combining different viable mutations affecting skin pigmentation, but, unlike casper, the crystal mutant also lacked pigmentation in the retinal pigment epithelium, enabling optical access to the eye (Antinucci and Hindges, 2016) [7]. Currently, a range of transparent zebrafish are being used for imaging studies. The following are the main genes that are mutated for the generation of transparency in zebrafish: mitfa (nacre zebrafish line) eliminates melanophore development; slc45a2 (albino zebrafish line) disrupts melanin synthesis; mpv17 (roy orbison zebrafish line) encodes a mitochondrial gene mutation that results in the complete loss of iridophores (D’Agati et al., 2017) [43]. There are also zebrafish lines bearing any two or all three mutations (casper, mitfa, and mpv17) responsible for crystal pigmentation. Using these mutants, many recent in vivo studies have been successfully performed (Karlsson et al., 2001; Chen et al., 2018; Auer et al., 2018; Kline et al., 2019) [23,44,45,46]. However, zebrafish are normally transparent only during the larval period and become opaque as they mature, mainly because of light scattering at lipid–water interfaces. Similar genetic manipulations were used to obtain a transparent mutant of the medaca fish. This mutant fish is very small and is relatively transparent, even in adult stages, allowing visualization of maturation of the gonads (Wakamatsu, 2001) [47]. Even in this case, light scattering is the limiting factor for transparency.

## 3. Methods for Reducing Light Scattering

### 3.1. Chemical Reduction of Scattering In Vivo

Chemical methods to achieve reduced scattering are similar to the methods used post mortem for tissue clearing (reviewed in: Chung et al., 2013; Richardson, Lichtman, 2015) [48,49] but must be compatible with living organisms. Usually, cell organelles (except the nucleus with RI ≈1.36; Steelman et al., 2017) [50] have RIs in the range 1.38–1.45 (Zhang et al., 2017) [51], while the cytoplasm has RI ≈ 1.38 (Barer and Ross, 1952; Ross, 1954; Steelman et al., 2017) [50,52,53]. Addition of neutral, biocompatible, and soluble chemical substance (optical clearing agent, OCA) with a greater RI that can enter the cytoplasm and augment the RI to the level of individual cellular organelles can eliminate the mismatch. This option will reduce multiple internal reflections or refractions of light in cellular organelles, eliminating the light scattering by organelles and leading to cell transparency. In addition, there is a mismatch between interstitial fluid and the cell, which produces scattering at the cell surface. Administration of the immersion liquid with an RI greater than that of the tissue interstitial fluid (as well as hyperosmotic properties) induces a partial replacement of the interstitial fluid by the immersion substance and hence decreases scattering. As immersion substances for in vivo clearing, aqueous solutions of glucose and mannitol, propylene glycol, glycerol, and other biocompatible chemicals may be used (Tuchin, 2006) [54].

Injection of the bioneutral propylene glycol (RI 1.43 at 589 nm) or glycerol (RI 1.47 at 589 nm) in vivo significantly reduces skin scattering and augments transparency (Tuchin et al., 1997; Tuchin, 2006) [54,55]. It was found that 75% glycerol had a significant clearing effect but caused local skin edema within 24 h, while 30% glycerol reduced the reflectance spectra of skin without injury. Moreover, 40% glucose injection produced clearing without necrosis in human skin (reviewed in: Wen et al., 2010; Zhu et al., 2013) [56,57]. It is sometimes beneficial to use membrane-penetrating agents, such as thiazone, together with the OCA, allowing faster intracellular OCA penetration by simple spreading of the mixture on the skin without injection (Shi et al., 2017) [58]. Similarly, Triton X-100 detergent can also be used with skin for these purposes (unpublished data). A special microneedle array can be used for OCA delivery to the skin (Tuan-Mahmood et al., 2013) [59]. For the purposes of super-resolution microscopy, an immersion substance with higher RI may be used to match the RIs of tissues to that of immersion oil (1.518), thus minimizing both light scattering and spherical aberrations in high-index objectives. For example, Intralipid emulsion (20%, Sigma-Aldrich) was used for in vivo super-resolution imaging of neuronal structures in mouse brain (Urban et al., 2018) [60]. This approach is similar to the SeeDB2 effect that enabled super-resolution microscopy of various tissue samples up to a depth of >100 μm (Ke et al., 2016) [61].

### 3.2. Genetic Approach to Reducing Scattering

As we already mentioned, there is an RI mismatch between the cytoplasm and cellular organelles as well as between the cells and the interstitial fluid, which produces optical scattering by living tissue, and an OCA delivered to the cytoplasm can reduce this mismatch. However, instead of delivering an external synthetic OCA to the tissue, it can be synthetized by the cell itself. The question arises: What endogenous OCAs are used in nature to make animal tissues transparent (for example, the transparent tissue in the eye), and how we can genetically manipulate and concentrate this substance in the cell? Currently, a majority of published studies have tried to first identify the natural OCA and its corresponding synthesis genes so that a genetic approach can be applied, and there are two main molecular classes of endogenous OCAs that have been identified.

#### 3.2.1. Glycosaminoglycans and Antifreeze Proteins in Fish

Many small water-borne animals, such as fish larvae, are transparent in order to survive natural selection. For example, eel (Leptocephali), surgeonfish (Acanthuridae) have very transparent larvae, and some larger fish, such as barreleye fish (Macropinna microstoma), develop a transparent shield on their head, including part of the cranium, which remains transparent in adulthood (Robison and Reisenbichler, 2008) [62]. It was proposed that these fish are transparent as a result of their bodies having energy storage in the form of transparent inert materials that consist primarily of glycosaminoglycan (GAG) compounds matrix with overall RI about 1.43 (Pfeiler 1988; Pfeiler et al. 2002; Miller, 2009) [63,64,65]. The gelatinous extracellular matrix of GAG comprises most of the bodies of the leptocephali, and this matrix contains a high percentage of water, ranging from 90–95% of total body weight (Miller, 2009) [65]. Pfeiler and coworkers (2002) [64] reported that hyaluronan is the principal GAG in the body matrix of seven species of anguilliform and in ladyfish (Elops saurus) and other Elopiformes leptocephali, but it is a minor GAG component in the bonefish *Albula* sp. (Albuliformes). In bonefish leptocephali, keratin sulfate appears to be the dominant form of GAG, with hyaluronan and chondroitin sulfate as minor components (Pfeiler et al. 1988) [63]. Thus, GAG in fish larvae bodies resembles the substance predominant in the vitreous humor in many vertebrates. Another OCA was determined after fish and bacterial antifreeze proteins (AFP) were discovered (DeVries and Wohlschlag, 1969; Davies et al., 2002) [66,67]. Present in the blood and inside cells, these proteins with ice-binding properties block ice formation at low temperatures. In addition, it was shown that their concentration inside the cell reduces light scattering, making cells more transparent (Gilbert et al., 2004) [68].

#### 3.2.2. Crystallins

Cells in the vertebrate optical tract are overwhelmingly transparent. Specific small-molecular-weight soluble proteins called crystallins (because of the crystal lens) are found in particularly high abundance in the cytoplasm of many cells of the vertebrate optical tract. Large quantities of crystallins were discovered in the cytoplasm of corneal cells, both in corneal epithelial cells and in stromal keratocytes (Krishnan et al., 2007; Jester, 2008) [69,70]. Similarly, crystallins are abundant in the cytoplasm of highly elongated fiber cells in the vertebrate lens and in lens epithelial cells (Delaye and Tardieu, 1983; Horwitz et al., 1999; Andley et al., 2009) [71,72,73]. In the retina, crystallins with a 23-kDa molecular weight (corresponding to both αA- and αB-crystallin) were described in frog (anuran) Müller cells (Simirskiĭ et al., 2003) [74], and αA-crystallin was found in the photoreceptor cells of mice and rats (Deretic et al., 1994; Maeda et al., 1999) [75,76]. Recently we described crystalline α, and specifically αA-crystallin, inside rat Muller cells and photoreceptors (Zayas-Santiago et al., 2018) [11].

It has been shown that crystallins are important for cell transparency in both the cornea and the lens (Delaye and Tardieu, 1983; Takemoto, Boyle, 1998; Jester, 2008) [70,71,77]. Chemical or genetic modification of crystallins leads to opacity, while reduction of soluble α-crystallins leads to cataracts (Datiles et al., 2008) [78]. Similarly, in the cornea, decreased expression of corneal crystallins in stromal keratocytes has been associated with increased in vitro and in vivo light scattering (Jester, 2008) [70]. In the lens, point mutations have been identified in the genes encoding α-, β-, and γ-crystallins that lead to the development of an hereditary form of human cataracts, either present at birth or developing at an early age (Graw, 2009; Andley et al., 2009) [73,79].

**Figure 1:**
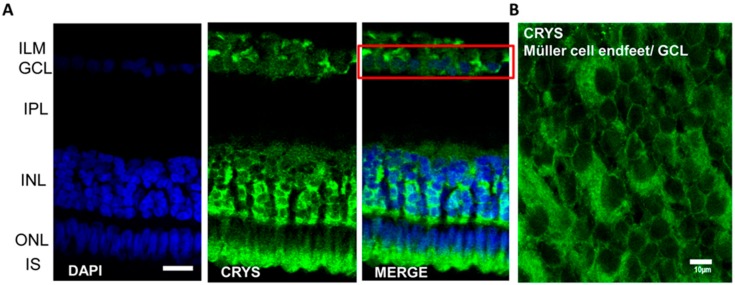
Immunolocalization of αA-crystallin in the caiman retina. (**A**) αA-crystallin (CRYS, green) is expressed in the inner nuclear layer (INL) surrounding the nuclei of neurons (DAPI nuclear staining, blue). Crystallin is also localized surrounding ganglion cell nuclei (red box) in the ganglion cell layer and in the inner segment area of photoreceptors. (**B**) αA-crystallin observed from the top of whole-retina tissue. Crystallin is confined to the endfeet of Müller cells in the ganglion cell layer (white arrow). IPL, inner plexiform layer; ILM, inner limiting membrane; GCL, ganglion cell layer; INL, inner nuclear layer; ONL, outer nuclear layer; IS, inner segments of photoreceptors. Scale bar in A, 20 μm and in B, 10 μm.

Crystallins have been found inside rat Muller cells and photoreceptors (Zayas-Santiago et al., 2018) [11]. Müller cells have been described as living light guides in the retina, which help to trap, hold, and conduct light from their endfeet to the photoreceptors (Franze et al., 2007; Reichenbach and Bringmann, 2010; Agte et al., 2011, Reichenbach and Bringmann, 2013; Agte et al., 2018; Karl et al.; 2018) [80,81,82,83,84,85]. Crystallins provide a possible mechanism for creating an ideal refractive medium for light transport. This colocalization of crystallin within Müller cells has not only been found in rats, but also in cats (Lewis et al., 1988) [86], frogs (Simirskii et al., 2003) [74], mice, and humans (Ruebsam et al., 2018) [87] as well as in caiman retina (previously unreported, Figure 1). Crystallin α is represented by three genes encoding crystallin αA (Cryaa) and crystallin αB (including Cryaba and Cryabb). In adult zebrafish Cryaa is not expressed in the skin, and there is mainly Cryz and Cryl1 expression and a small amount of Cryabb, Crybb1l2, Cryba4, Crybb3, Cxrym, and Cryba2b expression. In zebrafish embryos (according to the site: www. zfin.org), Cryaa in embryos is expressed only in the crystal lens, while Cryabb is distributed throughout the body. The expression of Cryaa mainly in the optical tract in all transparent tissues, including the retina, specifically implicates this αA crystallin in transparency also in caiman. In the caiman (Caiman crocodilus fuscus) retina, Cryaa expression can be found in the ganglion cell layer, the inner nuclear layer, and in the area of the photoreceptor inner segments (Figure 1A). In the inner nuclear layer, the Cryaa protein product, αA-crystallin, is observed surrounding the neuronal nuclei and forms structures around repetitive columns of neurons. These structures resemble the Müller cell body, similarly as it was observed using the classical glial marker vimentin and glutamine synthetase (Zayas-Santiago et al., 2014 [88], Figures 2B and 6A–C). In a retinal wholemount (Figure 1B), αA-crystallin is also observed in the ganglion cell layer and within the inner limiting membrane layer surrounding the ganglion cell nuclei. The islands formed correspond to the area covered by the Müller cell endfeet. αA-crystallin is also observed on top of the outer nuclear layer. The inner segments of photoreceptors are present in this area (Zayas-Santiago et al. 2014 [88], Figure 6A). These images were obtained following the same methodology discussed in Zayas-Santiago et al. (2018) [11] and were carried out under Institutional Animal Care and Use Committee approval and in accordance with the Association for Research in Vision and Ophthalmology Statement for the Use of Animals in Ophthalmic and Vision Research. Determining the possible role of crystallins in the transparency of the retina and in light guidance by Müller cells still needs to be investigated.

The involvement of αA-crystallin in transparency can be proven in microorganisms as well. It is known that Mycobacterium may have either a transparent (stationary)- or opaque (growing)-phase cell type, which can be transitioned from one to another without mutation, and this transition is temperature sensitive. The transparent variant tolerates higher growth temperatures and is resistant to most therapeutic agents. Because it is possible that the transparent variant is the causative agent of disease in man, it is of importance to understand the mechanism of the transparent-to-opaque transition (Woodley, David, 1976) [89]. αA-crystallins are both heat-shock proteins, allowing cells to survive at higher temperatures, and transparency-promoting agents, and it is possible that crystallins are the cause of the transparent-to-opaque transition. It is known that the 16-kDa α-crystallin homologue in Mycobacterium is the dominant protein produced by stationary-phase cultures in vitro, but it is undetectable in logarithmically growing cultures (Yuan et al., 1998; Stewart et al., 2006) [90,91].

#### 3.2.3. Possible Genetic Manipulation to Induce Transparency and Some Future Perspective

The use of glycosaminoglycans as a genetically manipulated OCA is difficult, and their effects are not entirely understood. On the other hand, αA-crystallin and its encoding gene is well characterized. While there is no active development in this direction currently, we suggest that the genetic approach has a promising future.

One difficulty is that crystallins have other functions in the organism, such as serving as heat-shock proteins and regulatory proteins, and their overexpression needs to be optimized for timing, intensity, and tissue specificity in order to minimize disturbances in organism development and performance. The overexpression approach has a long history of application in transgenic animals, as overexpression of additional copies of the target gene, maintained under control of a tetracycline-responsive promoter or by expression of the transactivator Cas9. The most physiologically adequate variant is considered to be conditional overexpression, which proceeds only in the presence of the inducer and does not interfere with normal development. Thus, mutated animals develop normally, transparency is induced only before the experiment, and the degree of transparency may be optimized for each case.

Transactivation of gene expression is a capability of the CRISPR-Cas9 gene-editing system (Perez-Pinera et al., 2013) [92]. It utilizes the fusion of inactivated (“dead”) Cas9 (which is able to form a complex only with its guide RNA and bind to the target, but is unable to cut the target) and a transcription activation domain, usually taken from a viral gene. This approach has the advantage of expressing the animal’s endogenous gene, whose level is determined by the basal level of gene expression and may be regulated by the addition of activation domains fused with Cas9 (Cheng et al., 2013) [93].

An experimental organism that could be used for this approach is zebrafish, which is already transparent in larval form but lacks the necessary transparency in adults, due to light scattering. Therefore, the goal of inducing crystallin transparency in adult zebrafish may be achieved by two strategies: (1) insertion of the crystallin genes under the control of tetracycline-responsive promoters and (2) insertion of the transactivator Cas9 gene with several genes for single-guide RNAs (sgRNAs). Both approaches have their inherent advantages and disadvantages, which may become obstacles to stable cell line generation. Moreover, this work will allow comparison of these approaches, which will also make it methodologically valuable.

Skin transparency may be achieved by transduction (i.e., infection) of all living skin cells with lentiviral vectors encoding αA-crystallin under a ubiquitous promotor. The efficiency of lentiviral infection was shown to be high with intradermal injections in mice (Woodley et al., 2004) [94], as lentiviruses are able to successfully infect non-dividing cells (and thus eliminate the need for creating a population of dividing cells, for example, by wounding). This approach requires cloning of the human αA-crystallin gene into the pLOC plasmid (widely used for lentiviral expression) and assembling it into viral particles with the accessory plasmids pdelta8.9 (packaging) and pVSVG (envelope). As a result, this packaging system generates replication-defective lentiviral particles so that these viruses are able to infect cells only once, without generation of viral offspring and thus maintaining system safety. At the same time, lentiviruses insert their genomes into the genomes of the host cells, thus achieving stable expression without the risk of plasmid elimination. The only disadvantage of lentiviral vectors is that they can tolerate insertion of only short genes. Fortunately, the αA-crystallin coding sequence is only 522 base pairs, so lentiviral capacity will not be a problem in this case. Also, by varying the multiplicity of infection (i.e., the number of infectious viral particles per cell), we have the opportunity to adjust the level of crystallin overexpression to non-toxic and effective levels. Moreover, this approach can be extended to practically any other tissue.

## 4. In Vivo Transparency of Bones and Other Mineralized Tissue

There are two types of bone tissue, cortical bone and cancellous (spongy) bone, and three types of mineralized dental tissue, including enamel, dentin, and cementum. All of these tissues are heavily mineralized and usually non-transparent, and light scattering is the main obstacle to their transparency. However, some animals naturally develop transparent bone that is highly mineralized. Many fish larvae have transparent bones, and part of the cranium of barreleye fish remains transparent during their lifetime, but the underlying physical principles are poorly understood (Robison and Reisenbichler, 2008) [62]. It was shown that the heavily mineralized teeth of the deep-sea dragonfish remain unusually transparent (in order to optically trick their prey), as they have special nanostructured dentin consisting of a woven pattern of nanometer rods (5 nm in diameter with 0.8 nm spacing) that nearly eliminates light scattering (Velasco-Hogan et al., 2019) [95]. This may be the key to engineering bone clearing in vivo—to discover how it happens naturally in fish. But for practical purposes, when it comes to tissue clearing, mineralization poses a particular challenge, because conventional removal of minerals using histological methods does not work for in vivo imaging, as these clearing substances are usually toxic (Greenbaum et al., 2017; Jing et al., 2019) [96,97]. A few articles have described the injection of OCAs to increase bone transparency. A decrease in absorption by up to 20% and a decrease in the skull bone scattering coefficient by up to 30% using glycerol have been demonstrated. Moreover, a decrease in bone reflectance by up to 70% was achieved using propylene glycol (Genina et al., 2008) [98]. Even so, the majority of experiments with optical imaging of neurons and blood vessels, for the purposes of optogenetics or optical stimulation/inhibition of neurons through the intact skull, used modified bone-thinning methods. In these methods a part of the skull bone was made significantly thinner by mechanical removal of the external bone surface, which was replaced with an artificial transparent material (e.g., glass, silicone, acrylic, or cyanoacrylate,) to form a window into the brain (Guo et al., 2014; Kalchenko et al., 2014; Steinzeig et al., 2017) [99,100,101]. As far as we know, there has been no genetics research to develop transgenic animals with naturally transparent crania (e.g., with reduced mineralization) for experimental studies.

## 5. Other Models of Transparency

Alternative views about transparency mechanisms have been recently developed. These are based on the importance of specific filament-like biological nanostructures, known as beaded intermediate filaments, in light propagation, because many chemical or genetic manipulations of these molecules lead to opacity (Quinlan et al., 1996) [102]. These filaments were found first in lens cells and in the cornea but have also been described in retinal cells (reviewed in: Zueva et al., 2016 [18]). Many researchers view these filaments as cytoskeletal elements that are only important for transparency because they maintain short-range order in the organization of cytoplasmic proteins and organelles, thus enabling light propagation (Tardieu and Delaye, 1988; Zayas-Santiago et al., 2018) [11,13]. An alternative explanation is that these specialized intermediate filaments decorated by αA-crystallin span the cell and can transmit light energy using quantum confinement mechanisms (Zueva et al., 2014; Zueva et al., 2016; Makarov et al., 2017) [18,103,104]. In this model, light scattering by organelles and protein particles is of no importance, because the light energy is contained inside the filaments during light propagation. These new models are intriguing, but still need substantial experimental confirmation and further development of methodological approaches to manipulate with.

## 6. Conclusions

Many investigators have developed methods that exploit the advantages of in vivo tissue transparency. Conventional in vivo imaging and spectroscopy, optical neural activity recording and stimulation, as well as optogenetics have all benefited from increased imaging depth using different methods of optical tissue clearing in vivo. There are two main approaches, chemical (pharmacological) and genetic, that reduce both light absorption and scattering. The power of both approaches has been demonstrated, but to completely eliminate the damaging effects of artificial OCAs, we suggest that genetic modification to obtain model organisms with transparent tissues represents the future of in vivo imaging.

## References

[1] Zylinski S., Johnsen S. (2011). Mesopelagic cephalopods switch between transparency and pigmentation to optimize camouflage in the deep. Curr. Biol..

[2] Zylinski S., Osorio D., Johnsen S. (2016). Cuttlefish see shape from shading, fine-tuning coloration in response to pictorial depth cues and directional illumination. Proc. Biol. Sci..

[3] Fetcho J.R., O’Malley D.M. (1995). Visualization of active neural circuitry in the spinal cord of intact zebrafish. J. Neurophysiol..

[4] O’Malley D.M., Zhou Q., Gahtan E. (2003). Probing neural circuits in the zebrafish: A suite of optical techniques. Methods.

[5] White R.M., Sessa A., Burke C., Bowman T., LeBlanc J., Ceol C., Bourque C., Dovey M., Goessling W., Burns C.E. (2008). Transparent adult zebrafish as a tool for in vivo transplantation analysis. Cell Stem Cell.

[6] Bin J.M., Lyons D.A. (2016). Imaging Myelination In Vivo Using Transparent Animal Models. Brain Plast..

[7] Antinucci P., Hindges R. (2016). A crystal-clear zebrafish for in vivo imaging. Sci. Rep..

[8] Harrison N.R., Laroche F.J., Gutierrez A., Feng H. (2016). Zebrafish Models of Human Leukemia: Technological Advances and Mechanistic Insights. Adv. Exp. Med. Biol..

[9] Nicolson T. (2017). The genetics of hair-cell function in zebrafish. J. Neurogenet..

[10] Saleem S., Kannan R.R. (2018). Zebrafish: An emerging real-time model system to study Alzheimer’s disease and neurospecific drug discovery. Cell Death Discov..

[11] Zayas-Santiago A., Ríos D.S., Zueva L.V., Inyushin M.Y. (2018). Localization of αA-Crystallin in Rat Retinal Müller Glial Cells and Photoreceptors. Microsc. Microanal..

[12] Brunsting A., Mullaney F. (1974). Differential light scattering from spherical mammalian cells. Biophys. J..

[13] Tardieu A., Delaye M. (1988). Eye lens proteins and transparency: From light transmission theory to solution X-ray structural analysis. Ann. Rev. Biophys. Chem..

[14] Jacques S.L., Pogue B.W. (2008). Tutorial on diffuse light transport. J. Biomed. Opt..

[15] Jacques S.L. (2013). Optical properties of biological tissues: A review. Phys. Med. Biol..

[16] Perelman L.T., Yoshizawa T. (2017). Light Scattering. Handbook of Optical Metrology: Principles and Applications.

[17] Jansen E.D., Pickett P.M., Mackanos M.A., Virostko J. (2006). Effect of optical tissue clearing on spatial resolution and sensitivity of bioluminescence imaging. J. Biomed. Opt..

[18] Zueva L., Golubeva T., Korneeva E., Makarov V., Khmelinskii I., Inyushin M. (2016). Foveolar Müller Cells of the Pied Flycatcher: Morphology and Distribution of Intermediate Filaments Regarding Cell Transparency. Microsc. Microanal..

[19] Xu J., Ruan H., Liu Y., Zhou H., Yang C. (2017). Focusing light through scattering media by transmission matrix inversion. Opt. Express.

[20] Cua M., Zhou H., Yang C. (2017). Imaging moving targets through scattering media. Opt. Express.

[21] Parvez S., Kang M., Chung H.S., Cho C., Hong M.C., Shin M.K., Bae H. (2006). Survey and mechanism of skin depigmenting and lightening agents. Phytother. Res..

[22] Heriniaina R.M., Dong J., Kalavagunta P.K., Wu H.L., Yan D.S., Shang J. (2018). Effects of six compounds with different chemical structures on melanogenesis. Chin. J. Nat. Med..

[23] Karlsson J., von Hofsten J., Olsson P.E. (2001). Generating transparent zebrafish: A refined method to improve detection of gene expression during embryonic development. Mar. Biotechnol..

[24] Rebelo J., Auerbach G., Bader G., Bracher A., Nar H., Hösl C., Schramek N., Kaiser J., Bacher A., Huber R. (2003). Biosynthesis of Pteridines. Reaction Mechanism of GTP Cyclohydrolase I. J. Mol. Biol..

[25] Matthews S.J., Ross N.W., Lall S.P., Gill T.A. (2006). Astaxanthin binding protein in Atlantic salmon. Comp. Biochem. Physiol. B Biochem. Mol. Biol..

[26] Hata M., Hata M. (1973). Carotenoid pigments in goldfish (Carassius auratus L.). VI. The effect of ACTH and phenylthiourea on the discoloration and the carotenoid metabolism. Comp. Biochem. Physiol. A Comp. Physiol..

[27] Forrest H.S., Glassman E., Mitchell H.K. (1956). Conversion of 2-amino-4-hydroxypteridine to isoxanthopterin in D. Melanogaster. Science.

[28] Demski L.S. (1992). Chromatophore systems in teleosts and cephalopods: A levels oriented analysis of convergent systems. Brain Behav. Evol..

[29] Liu T.H., Chiao C.C. (2017). Mosaic Organization of Body Pattern Control in the Optic Lobe of Squids. J. Neurosci..

[30] Reiter S., Hülsdunk P., Woo T., Lauterbach M.A., Eberle J., Akay L.A., Longo A., Meier-Credo J., Kretschmer F., Langer J.D. (2018). Elucidating the control and development of skin patterning in cuttlefish. Nature.

[31] Saenko S.V., Teyssier J., van der Marel D., Milinkovitch M.C. (2013). Precise colocalization of interacting structural and pigmentary elements generates extensive color pattern variation in Phelsuma lizards. BMC Biol..

[32] Teyssier J., Saenko S.V., van der Marel D., Milinkovitch M.C. (2015). Photonic crystals cause active colour change in chameleons. Nat. Commun..

[33] Lythgoe J.N., Shand J., Foster R.G. (1984). Visual pigment in fish iridocytes. Nature.

[34] Schweikert L.E., Fitak R.R., Johnsen S. (2018). De novo transcriptomics reveal distinct phototransduction signaling components in the retina and skin of a color-changing vertebrate, the hogfish (Lachnolaimus maximus). J. Comp. Physiol. A Neuroethol. Sens. Neural Behav. Physiol..

[35] Ziegler I., McDonald T., Hesslinger C., Pelletier I., Boyle P. (2000). Development of the pteridine pathway in the zebrafish, Danio rerio. J. Biol. Chem..

[36] Eskova A., Chauvigné F., Maischein H.M., Ammelburg M., Cerdà J., Nüsslein-Volhard C., Irion U. (2017). Gain-of-function mutations in Aqp3a influence zebrafish pigment pattern formation through the tissue environment. Development.

[37] Gunia-Krzyżak A., Popiol J., Marona H. (2016). Melanogenesis Inhibitors: Strategies for Searching for and Evaluation of Active Compounds. Curr. Med. Chem..

[38] Lajis A.F.B. (2018). A Zebrafish Embryo as an Animal Model for the Treatment of Hyperpigmentation in Cosmetic Dermatology Medicine. Medicina (Kaunas).

[39] Ono K., Viet C.T., Ye Y., Dang D., Hitomi S., Toyono T., Inenaga K., Dolan J.C., Schmidt B.L. (2017). Cutaneous pigmentation modulates skin sensitivity via tyrosinase-dependent dopaminergic signalling. Sci. Rep..

[40] Rader K. (2004). Making Mice, Standardizing Animals for American Biomedical Research.

[41] Conway K.S., Trudeau J. (2018). Sunshine, fertility and racial disparities. Econ. Hum. Biol..

[42] Lister J.A., Robertson C.P., Lepage T., Johnson S.L., Raible D.W. (1999). Nacre Encodes a Zebrafish Microphthalmia-Related Protein that Regulates Neural-Crest-Derived Pigment Cell Fate. Development.

[43] D’Agati G., Beltre R., Sessa A., Burger A., Zhou Y., Mosimann C., White R.M. (2017). A defect in the mitochondrial protein Mpv17 underlies the transparent casper zebrafish. Dev. Biol..

[44] Chen Z., Chou S.W., McDermott B.M. (2018). Ribeye protein is intrinsically dynamic but is stabilized in the context of the ribbon synapse. J. Physiol..

[45] Auer F., Vagionitis S., Czopka T. (2018). Evidence for Myelin Sheath Remodeling in the CNS Revealed by In Vivo Imaging. Curr. Biol..

[46] Kline T.L., Sussman C.R., Irazabal M.V., Mishra P.K., Pearson E.A., Torres V.E., Macura S.I. (2019). Three-dimensional NMR microscopy of zebrafish specimens. NMR Biomed..

[47] Wakamatsu Y., Pristyazhnyuk S., Kinoshita M., Tanaka M., Ozato K. (2001). The see-through medaka: A fish model that is transparent throughout life. Proc. Natl. Acad. Sci. USA.

[48] Chung K., Wallace J., Kim S.Y., Kalyanasundaram S., Andalman A.S., Davidson T.J., Mirzabekov J.J., Zalocusky K.A., Mattis J., Denisin A.K. (2013). Structural and molecular interrogation of intact biological systems. Nature.

[49] Richardson D.S., Lichtman J.W. (2015). Clarifying Tissue Clearing. Cell.

[50] Steelman Z.A., Eldridge W.J., Weintraub J.B., Wax A. (2017). Is the nuclear refractive index lower than cytoplasm? Validation of phase measurements and implications for light scattering technologies. J. Biophotonics.

[51] Zhang Q., Zhong L., Tang P., Yuan Y., Liu S., Tian J., Lu X. (2017). Quantitative refractive index distribution of single cell by combining phase-shifting interferometry and AFM imaging. Sci. Rep..

[52] Barer R., Ross K.A. (1952). Refractometry of living cells. J. Physiol..

[53] Ross K.F.A. (1954). Measurement of the Refractive Index of Cytoplasmic Inclusions in Living Cells by the Interference Microscope. Nature.

[54] Tuchin V.V. (2006). Optical Clearing of Tissues and Blood.

[55] Tuchin V.V., Maksimova I.L., Zimnyakov D.A., Kon I.L., Mavlyutov A.H., Mishin A.A. (1997). Light propagation in tissues with controlled optical properties. J. Biomed. Opt..

[56] Wen X., Mao Z., Han Z., Tuchin V.V., Zhu D. (2010). In vivo skin optical clearing by glycerol solutions: Mechanism. J. Biophoton..

[57] Zhu D., Larin K.V., Luo Q., Tuchin V.V. (2013). Recent progress in tissue optical clearing. Laser Photon. Rev..

[58] Shi R., Guo L., Zhang C., Feng W., Li P., Ding Z., Zhu D. (2017). A useful way to develop effective in vivo skin optical clearing agents. J. Biophoton..

[59] Tuan-Mahmood T.M., McCrudden M.T., Torrisi B.M., McAlister E., Garland M.J., Singh T.R., Donnelly R.F. (2013). Microneedles for intradermal and transdermal drug delivery. Eur. J. Pharm. Sci..

[60] Urban B.E., Xiao L., Chen S., Yang H., Dong B., Kozorovitskiy Y., Zhang H.F. (2018). In Vivo Superresolution Imaging of Neuronal Structure in the Mouse Brain. IEEE Trans. Biomed. Eng..

[61] Ke M.T., Nakai Y., Fujimoto S., Takayama R., Yoshida S., Kitajima T.S., Sato M., Imai T. (2016). Super-Resolution Mapping of Neuronal Circuitry With an Index-Optimized Clearing Agent. Cell Rep..

[62] Robison B.H., Reisenbichler K.R. (2008). Macropinna microstoma and the paradox of its tubular eyes. Copeia.

[63] Pfeiler E. (1988). Isolation and partial characterization of a novel keratan sulfate proteoglycan from metamorphosing bonefish (Albula) larvae. Fish Physiol. Biochem..

[64] Pfeiler E., Toyoda H., Williams M.D., Nieman R.A. (2002). Identification, structural analysis and function of hyaluronan in developing fish larvae (leptocephali). Comp. Biochem. Physiol. B Biochem. Mol. Biol..

[65] Miller M.J. (2009). Ecology of Anguilliform Leptocephali: Remarkable Transparent Fish Larvae of the Ocean Surface Layer. Aqua-BioSci. Monogr..

[66] DeVries A.L., Wohlschlag D.E. (1969). Freezing resistance in some Antarctic fishes. Science.

[67] Davies P.L., Baardsnes J., Kuiper M.J., Walker V.K. (2002). Structure and function of antifreeze proteins. Philos. Trans. R. Soc. Lond. B Biol. Sci..

[68] Gilbert J.A., Hill P.J., Dodd C.E., Laybourn-Parry J. (2004). Demonstration of antifreeze protein activity in Antarctic lake bacteria. Microbiology.

[69] Krishnan K., Kathiresan T., Raman R., Rajini B., Dhople V.M., Aggrawal R.K., Sharma Y. (2007). Ubiquitous lens alpha-, beta-, and gamma-crystallins accumulate in anuran cornea as corneal crystallins. J. Biol. Chem..

[70] Jester J.V. (2008). Corneal crystallins and the development of cellular transparency. Semin. Cell Dev. Biol..

[71] Delaye M., Tardieu A. (1983). Short-range order of crystallin proteins accounts for eye lens transparency. Nature.

[72] Horwitz J., Bova M.P., Ding L.L., Haley D.A., Stewart P.L. (1999). Lens alpha-crystallin: Function and structure. Eye.

[73] Andley U.P. (2009). Effects of alpha-crystallin on lens cell function and cataract pathology. Curr. Mol. Med..

[74] Simirskiĭ V.N., Panova I.G., Sologub A.A., Aleĭnikova K.S. (2003). Localization of crystallins in Muellerian cells in the grass frog retina. Ontogenez.

[75] Deretic D., Aebersold R.H., Morrison H.D., Papermaster D.S. (1994). Alpha A- and alpha B-crystallin in the retina. Association with the post-Golgi compartment of frog retinal photoreceptors. J. Biol. Chem..

[76] Maeda A., Ohguro H., Maeda T., Nakagawa T., Kuroki Y. (1999). Low expression of alphaA-crystallins and rhodopsin kinase of photoreceptors in retinal dystrophy rat. Investig. Ophthalmol. Vis. Sci..

[77] Takemoto L., Boyle D. (1998). The possible role of alpha-crystallins in human senile cataractogenesis. Int. J. Biol. Macromol..

[78] Datiles M.B., Ansari R.R., Suh K.I., Vitale S., Reed G.F., Zigler J.S., Ferris F.L. (2008). Clinical detection of precataractous lens protein changes using dynamic light scattering. Arch. Ophthalmol..

[79] Graw J. (2009). Genetics of crystallins: Cataract and beyond. Exp. Eye Res..

[80] Franze K., Grosche J., Skatchkov S.N., Schinkinger S., Foja C., Schild D., Uckermann O., Travis K., Reichenbach A., Guck J. (2007). Muller cells are living optical fibers in the vertebrate retina. Proc. Natl. Acad. Sci. USA.

[81] Reichenbach A., Bringmann A. (2010). Müller Cells in the Healthy and Diseased Retina.

[82] Agte S., Junek S., Matthias S., Ulbricht E., Erdmann I., Wurm A., Schild D., Käs J.A., Reichenbach A. (2011). Müller glial cell-provided cellular light guidance through the vital guinea-pig retina. Biophys. J..

[83] Reichenbach A., Bringmann A. (2013). New functions of Müller cells. Glia.

[84] Agte S., Savvinov A., Karl A., Zayas-Santiago A., Ulbricht E., Makarov V.I., Reichenbach A., Bringmann A., Skatchkov S.N. (2018). Müller glial cells contribute to dim light vision in the spectacled caiman (Caiman crocodilus fuscus): Analysis of retinal light transmission. Exp. Eye Res..

[85] Karl A., Agte S., Zayas-Santiago A., Makarov F.N., Rivera Y., Benedikt J., Francke M., Reichenbach A., Skatchkov S.N., Bringmann A. (2018). Retinal adaptation to dim light vision in spectacled caimans (Caiman crocodilus fuscus): Analysis of retinal ultrastructure. Exp. Eye Res..

[86] Lewis G.P., Erickson P.A., Kaska D.D., Fisher S.K. (1988). An immunocytochemical comparison of Müller cells and astrocytes in the cat retina. Exp. Eye Res..

[87] Ruebsam A., Dulle J.E., Myers A.M., Sakrikar D., Green K.M., Khan N.W., Schey K., Fort P.E. (2018). A specific phosphorylation regulates the protective role of αA-crystallin in diabetes. JCI Insight.

[88] Zayas-Santiago A., Agte S., Rivera Y., Benedikt J., Ulbricht E., Karl A., Dávila J., Savvinov A., Kucheryavykh Y., Inyushin M. (2014). Unidirectional photoreceptor-to-Müller glia coupling and unique K+ channel expression in Caiman retina. PLoS ONE.

[89] Woodley C.L., David H.L. (1976). Effect of temperature on the rate of the transparent to opaque colony type transition in Mycobacterium avium. Antimicrob. Agents Chemother..

[90] Yuan Y., Crane D.D., Simpson R.M., Zhu Y.Q., Hickey M.J., Sherman D.R., Barry C.E. (1998). The 16-kDa alpha-crystallin (Acr) protein of Mycobacterium tuberculosis is required for growth in macrophages. Proc. Natl. Acad. Sci. USA.

[91] Stewart J.N., Rivera H.N., Karls R., Quinn F.D., Roman J., Rivera-Marrero C.A. (2006). Increased pathology in lungs of mice after infection with an alpha-crystallin mutant of Mycobacterium tuberculosis: changes in cathepsin proteases and certain cytokines. Microbiology.

[92] Cheng A.W., Wang H., Yang H., Shi L., Katz Y., Theunissen T.W., Rangarajan S., Shivalila C.S., Dadon D.B., Jaenisch R. (2013). Multiplexed activation of endogenous genes by CRISPR-on, an RNA-guided transcriptional activator system. Cell Res..

[93] Perez-Pinera P., Kocak D.D., Vockley C.M., Adler A.F., Kabadi A.M., Polstein L.R., Thakore P.I., Glass K.A., Ousterout D.G., Leong K.W. (2013). RNA-guided gene activation by CRISPR-Cas9-based transcription factors. Nat. Methods.

[94] Woodley D.T., Keene D.R., Atha T. (2004). Intradermal injection of lentiviral vectors corrects regenerated human dystrophic epidermolysis bullosa skin tissue in vivo. Mol. Ther..

[95] Velasco-Hogan A., Deheyn D.D., Koch M., Nothdurft B., Arzt E., Meyers M.A. (2019). On the Nature of the Transparent Teeth of the Deep-Sea Dragonfish, Aristostomias scintillans. Matter.

[96] Greenbaum A., Chan K.Y., Dobreva T., Brown D., Balani D.H., Boyce R., Kronenberg H.M., McBride H.J., Gradinaru V. (2017). Bone CLARITY: Clearing, imaging, and computational analysis of osteoprogenitors within intact bone marrow. Sci. Transl. Med..

[97] Jing D., Yi Y., Luo W., Zhang S., Yuan Q., Wang J., Lachika E., Zhao Z., Zhao H. (2019). Tissue Clearing and Its Application to Bone and Dental Tissues. J. Dent. Res..

[98] Genina E.A., Bashkatov A.N., Tuchin V.V. (2008). Optical Clearing of Cranial Bone. Adv. Opt. Technol..

[99] Kalchenko V., Israeli D., Kuznetsov Y., Harmelin A. (2014). Transcranial optical vascular imaging (TOVI) of cortical hemodynamics in mouse brain. Sci. Rep..

[100] Guo Z.V., Li N., Huber D., Ophir E., Gutnisky D., Ting J.T., Feng G., Svoboda K. (2014). Flow of Cortical Activity Underlying a Tactile Decision in Mice. Neuron.

[101] Steinzeig A., Molotkov D., Castrén E. (2017). Chronic imaging through “transparent skull” in mice. PLoS ONE.

[102] Quinlan R.A., Carte J.M., Sandilands A., Prescott A.R. (1996). The beaded filament of the eye lens: An unexpected key to intermediate filament structure and function. Trends Cell Biol..

[103] Zueva L., Makarov V., Zayas-Santiago A., Golubeva T., Korneeva E., Savvinov A., Eaton M., Skatchkov S., Inyushin M. (2014). Müller cell alignment in bird fovea: possible role in vision. J. Neurosci. Neuroeng..

[104] Makarov V., Zueva L., Golubeva T., Korneeva E., Khmelinskii I., Inyushin M. (2017). Quantum mechanism of light transmission by the intermediate filaments in some specialized optically transparent cells. Neurophotonics.

